# PTEN regulates AMPA receptor-mediated cell viability in iPS-derived motor neurons

**DOI:** 10.1038/cddis.2014.55

**Published:** 2014-02-27

**Authors:** D-J Yang, X-L Wang, A Ismail, C J Ashman, C F Valori, G Wang, S Gao, A Higginbottom, P G Ince, M Azzouz, J Xu, P J Shaw, K Ning

**Affiliations:** 1 East Hospital, Tongji University School of Medicine, Shanghai, China; 2 Department of Neuroscience, Sheffield Institute for Translational Neuroscience (SITraN), University of Sheffield, Sheffield, UK

**Keywords:** PTEN, iPSCs, motor neuron, AMPA receptor

## Abstract

Excitatory transmission in the brain is commonly mediated by the *α*-amino-3-hydroxy-5-methyl-4-isoxazole propionic acid (AMPA) receptors. In amyotrophic lateral sclerosis (ALS), AMPA receptors allow cytotoxic levels of calcium into neurons, contributing to motor neuron injury. We have previously shown that oculomotor neurons resistant to the disease process in ALS show reduced AMPA-mediated inward calcium currents compared with vulnerable spinal motor neurons. We have also shown that PTEN (phosphatase and tensin homolog deleted on chromosome 10) knockdown via siRNA promotes motor neuron survival in models of spinal muscular atrophy (SMA) and ALS. It has been reported that inhibition of PTEN attenuates the death of hippocampal neurons post injury by decreasing the effective translocation of the GluR2 subunit into the membrane. In addition, leptin can regulate AMPA receptor trafficking via PTEN inhibition. Thus, we speculate that manipulation of AMPA receptors by PTEN may represent a potential therapeutic strategy for neuroprotective intervention in ALS and other neurodegenerative disorders. To this end, the first step is to establish a fibroblast–iPS–motor neuron in *vitro* cell model to study AMPA receptor manipulation. Here we report that iPS-derived motor neurons from human fibroblasts express AMPA receptors. PTEN depletion decreases AMPA receptor expression and AMPA-mediated whole-cell currents, resulting in inhibition of AMPA-induced neuronal death in primary cultured and iPS-derived motor neurons. Taken together, our results imply that PTEN depletion may protect motor neurons by inhibition of excitatory transmission that represents a therapeutic strategy of potential benefit for the amelioration of excitotoxicity in ALS and other neurodegenerative disorders.

Amyotrophic lateral sclerosis (ALS) is a devastating neurodegenerative disorder affecting upper and lower motor neurons (MNs) and leading to death within 2–3 years from diagnosis. Between 90 and 95% of cases are sporadic in origin, whereas the remaining 5–10% of cases are familial. Of these, ∼20% carry mutations in the gene encoding the superoxide dismutase 1 enzyme (SOD1).^1^ Transgenic mice expressing mutant forms of human SOD1 are used as a model of familial ALS.^2^ We have defined the gene expression profiles of MNs isolated from the spinal cord of G93ASOD1 transgenic mice at different stages of disease, by combining the use of laser capture microdissection (LCM) and microarray technology. This work highlighted the involvement of metabolism in the first stages of disease, along with a substantial upregulation of transcription-related transcripts.^3^ Metabolic impairment suggested that astrocytes might also play a crucial role in the first stages of disease, supporting the results from other groups.^4, 5, 6^

Hexanucleotide GGGGCC intronic expansions in the newly identified *C9ORF72* (*chromosome 9 open reading frame 72*) gene represents the most common cause of both familial and sporadic ALS,^7, 8^ responsible for up to 50% of familial ALS and ∼10% of sporadic ALS, but to date *SOD1* mutations have been the genetic subtype most widely studied and utilized to model ALS. In the presence of mutant SOD1, multiple interacting factors contribute to MN injury including protein misfolding and aggregation, defective axonal transport, excitotoxicity, mitochondrial dysfunction, dysregulated transcription and RNA processing, endoplasmic reticulum stress, apoptosis, oxidative stress as well as toxicity caused by nonneuronal cells.^9^ Of these mechanisms, excitotoxicity is considered to play a key role. Routine excitatory transmission in the brain is predominantly mediated by *α*-amino-3-hydroxy-5-methyl-4-isoxazole propionic acid (AMPA) receptors. In ALS, there is a body of evidence that excitotoxicity mediated by calcium-permeable AMPA receptors contributes significantly to MN injury. Riluzole, an antiexcitotoxic agent, is the only drug proven to slow the disease progression in humans.^10^ AMPA receptors have been proposed to play a major role in the selective death of MNs in ALS. These characteristics are related to the way MNs handle Ca^2+^. MNs have a low Ca^2+^-buffering capacity and a high number of Ca^2+^-permeable AMPA receptors. A significantly different ratio between inhibitory and excitatory synapses was present in SOD1^G93A^ mouse spinal cord slice cultures. SOD1^G93A^ MNs exhibited increased vulnerability to AMPA glutamate receptor-mediated excitotoxic stress before the onset of an overt disease phenotype.^11^ The levels of the glutamate receptor 2 (GluR2) AMPA subunit, which plays an important role in the maintenance of calcium impermeability of AMPA receptors, are decreased in spinal MNs before symptom onset in conjunction with a modest increase of GluR3 expression in SOD1^G93A^ mice.^12^ GluR2 is a subunit of the AMPA receptor, and the adenosine for the Q/R site of its pre-mRNA is converted to inosine (A-to-I conversion) by the action of adenosine deaminase acting on RNA 2 (ADAR2). Failure of A-to-I conversion at this site affects multiple AMPA receptor properties, including the Ca^2+^ permeability of the receptor-coupled ion channel. Inefficient GluR2 Q/R site editing is a disease-specific molecular dysfunction reported in the MNs of sporadic ALS patients.^13^

We have previously shown that oculomotor neurons resistant to the disease process in ALS show reduced AMPA-mediated inward calcium currents, and higher GABA-mediated chloride currents, compared with spinal MNs that are vulnerable to the disease process.^14^ We have also demonstrated that knockdown of phosphatase and tensin homolog deleted on chromosome 10 (PTEN) via siRNA promotes MN survival in models of spinal muscular atrophy (SMA)^15^ and ALS.^16^ Inhibition of PTEN attenuates the death of hippocampal neurons post injury by decreasing the effective translocation of the GluR2 subunit into the membrane.^17^ Leptin promotes GluR1 trafficking to hippocampal synapses via PTEN inhibition.^18^ Furthermore, PI3-kinase that is negatively regulated by PTEN is required for maintaining AMPA receptor clustering at the postsynaptic membranes^19, 20^ and AMPA receptor surface expression.^21^ Thus, we speculate that manipulation of AMPA receptor expression and function by PTEN may represent a potential therapeutic strategy for clinical intervention in ALS. The breakthrough that human fibroblasts can be transformed into induced pluripotent stem (iPS) cells^22^ and these could then be differentiated into MNs^23^ opened new perspectives, providing a model of human origin for sporadic and familial cases of ALS. The aim of the present study is to establish a fibroblast-derived iPS-MN *in vitro* cell model for manipulation of AMPA receptor expression and function.

## Results

### Generation and characterization of human iPSCs (hiPSCs) from adult human dermal fibroblasts (HDFs)

**Figure 1:**
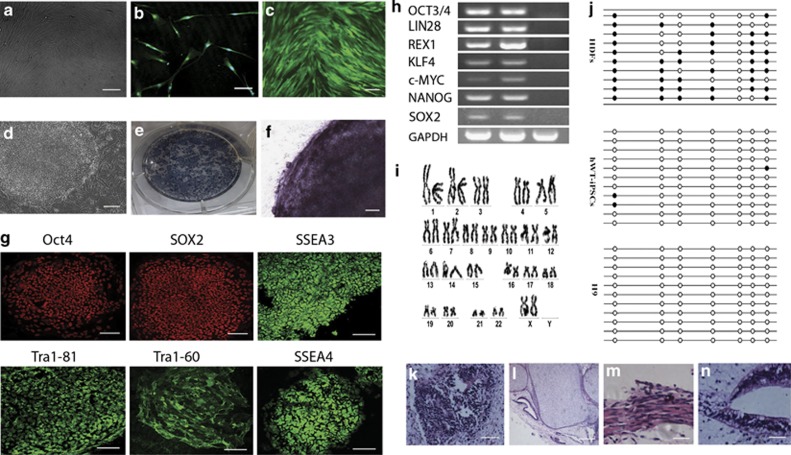
Characterization of human dermal fibroblasts used for hiPSC induction. (**a**) Culture of human dermal fibroblasts (bar=50 *μ*m). (**b**) TE-7 immunostaining confirmed that the resulting cultures were of fibroblast identity (bar=100 *μ*m). (**c**) Phase contrast image of human dermal fibroblasts transfected with GFP retroviruses (bar=100 *μ*m). (**d**) The hESC-like hiPS colony (bar=50 *μ*m). (**e** and **f**) These hESC-like colonies showed high alkaline phosphatase expression (bar=50 *μ*m). (**g**) Immunocytofluorescence of the hiPSCs showing the expression of multiple hESC markers (bar=100 *μ*m). (**h**) RT-PCR analysis of hESC marker genes in hiPSCs. (**i**) No karyotypic abnormalities were observed in the hiPSCs. (**j**) Open circles indicate unmethylated CpG dinucleotides, whereas closed circles indicate methylated CpGs in the promotor of Oct4. After transplantation into the hind limb muscle of NOD/SCID mice, hiPSCs generated teratomas showing (**k**) neural tissue (ectoderm; bar=100 *μ*m), (**l**) cartilage (mesoderm; bar=100 *μ*m), (**m**) muscle (mesoderm; bar=100 *μ*m) and (**n**) primitive gut (endoderm; bar=100 *μ*m)

Under human research subject and stem cell protocols as approved by the institutional review boards, we recruited a healthy 40-year-old Chinese female to donate a skin biopsy to be used in reprogramming studies and the production of pluripotent stem cell lines. Three other healthy controls were also used to generate induced pluripotent stem cells (iPSCs) under ethical approval in the United Kingdom. The HDFs were isolated and plated under standard fibroblast conditions (Figure 1a). After four passages, the fibroblast identity in the resulting cell lines was confirmed by TE-7 antibody staining (Figure 1b). The cells were then used for generating iPS cells after infection with retroviruses containing human OCT3/4, SOX2, C-MYC and KLF4. As a control, we introduced green fluorescent protein (GFP) into HDF with pMXs-GFP retrovirus produced in PLAT-A packaging cells. More than 80% of HDF treated in this way expressed GFP (Figure 1c). Using this method, the dish was nearly covered with >100 granulated colonies ∼25 days later. We observed distinct types of colonies that were flat and resembled hES cell-like colonies in between the granulated cells. At day 30, we picked 20 hES cell-like colonies and mechanically disaggregated them into small clumps without enzymatic digestion. The hES-like cells expanded on irradiated mouse embryonic fibroblasts (MEFs) using primate ES cell medium containing fibroblast growth factor-basic (bFGF).

To establish that reprogramming of the human fibroblasts had occurred, and that the putative hiPSCs were pluripotent, we evaluated their similarity to human embryonic stem cells (hESCs). Although we initially isolated 20 hiPSC clones, we focused on an in-depth characterization of only one of these clones. As illustrated in Figure 1d, the derived cells displayed the morphological characteristics of undifferentiated hESCs (i.e., large, compact, multicellular colonies of cells with a high nucleus-to-cytoplasm ratio) and showed high telomerase activity (Figures 1e and f). Moreover, they expressed several frequently used hESC markers (i.e., OCT3/4, SOX2, LIN28, NANOG, REX1) as shown by immunohistochemistry (Figure 1g) and reverse transcription-PCR (RT-PCR; Figure 1h). In addition, the putative hiPSC line also maintained a normal karyotype (Figure 1i). The colonies continued to passage after at least 6 months by passaging once a week. The split ratio was 1 : 3 to 1 : 6. Bisulfite genomic sequencing analyses evaluating the methylation status of cytosine guanine dinucleotides (CpG) in the promoter region of the pluripotent-associated gene Oct4 revealed that this was highly unmethylated, whereas the CpG dinucleotides of the region were highly methylated in parental HDF (Figure 1j). These findings indicate that this promoter is active in hiPSCs.

To test pluripotency *in vivo*, we transplanted human iPS cells subcutaneously into the hind limb muscle of NOD/SCID mouse. At 8 weeks after injection, we observed tumor formation. Histological examination showed that the tumor contained various tissues: neural tissues (ectoderm, Figure 1k), cartilage (mesoderm, Figure 1l), muscle (mesoderm, Figure 1m) and primitive gut (endoderm, Figure 1n).

Taken together, these data demonstrate that reprogramming of wild-type fibroblasts to a pluripotent state had successfully occurred.

### MN differentiation of hiPSCs

**Figure 2:**
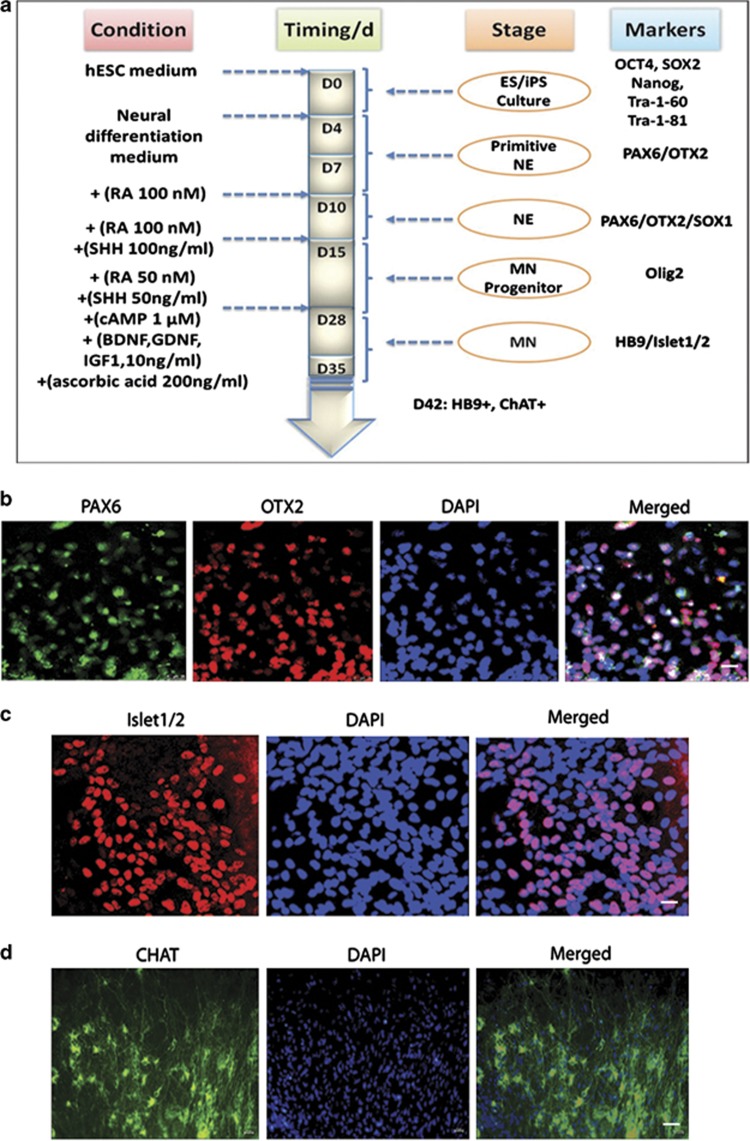
Generation and characterization of spinal motor neurons from hiPSCs. (**a**) Schematic drawing of the experimental setup and strategy to derive induced spinal motor neurons. (**b**) When differentiated for 12 days, the hiPSC-derived primitive neuroepithelial cells are positive for OTX2 (red) and PAX6 (green), bar=25 *μ*m. (**c**) When differentiated for 35 days, the hiPSC-derived motor neurons are positive for Islet 1/2 (red), bar=50 *μ*m. (**d**) When differentiated for 45 days, hiPSC-derived motor neurons become mature, and are positive for ChAT (green), bar=50 *μ*m. DAPI shown as blue denotes DAPI-stained nuclei

Several groups have devised protocols to differentiate hESCs/hiPSCs to functional MNs.^23, 24, 25^ Here we chose a protocol that follows the principles of normal development and leads to a high efficiency in the production of the target MNs (Figure 2a). We raised hiPSCs in co-culture with a MEF feeder layer, as previously described. The cultures were raised to 80–90% confluence and exhibited a uniform undifferentiated phenotype to achieve neuro-ectodermalization before inducing them to form embryoid bodies (EBs) in suspension cultures. EB aggregates are usually grown as free-floating spheres. The EBs were cultured for 7 days and then they were attached to tissue culture dishes to initiate the differentiation of neuroepithelial cells that soon emerge as neural epithelial rosettes. After 3 days, the primitive neural epithelial cells formed and expressed anterior transcription factors, such as PAX6 and OTX2 (Figure 2b). After an additional 5 days in the presence of retinoic acid (RA), definitive neuroepithelial cells tended to form neural tube-like rosettes. The neural rosettes were gently blown off and treated with both RA and sonic hedgehog (SHH), resulting in the appearance of MN progenitors. These cells then differentiated to spinal MNs in week 5 and expressed transcription factors, such as Islet1/2 (Figure 2c). This protocol generated ∼50% of Islet1/2-expressing MNs from the original hWT-iPSC cells. These MNs, when further cultured in the presence of neurotrophic factors, extend long axonal projections, express choline acetyltransferase (ChAT; Figure 2d) and become electrophysiologically active.

### PTEN knockdown increases phosphorylation of Akt and Bad in primary cultured MNs and iPS-derived MNs

**Figure 3:**
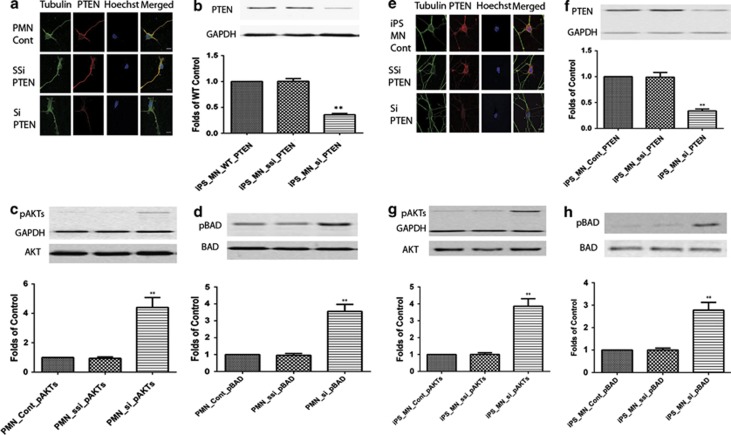
PTEN knockdown (**b** and **f**) increases phosphorylation of AKT (**c** and **g**) and Bad (**d** and **h**) in primary cultured motor neurons and iPS-derived motor neurons. Primary (**a**) or iPS-derived (**e**) motor neurons were immunostained with anti-tubulin (green), Hoechst (nuclear staining in blue) and PTEN (red). Scale bar, 20 *μ*m. Western blotting on homogenates from primary cultured (**b–d**) and iPS-derived motor neurons. PTEN is normalized by GAPDH (**b** and **f**) and pAKT is normalized by AKT (**c** and **g**). pBAD is normalized by BAD (**d** and **h**). Results represent the mean+S.E.M. from four independent experiments. **P<0.01, tested by one-way ANOVA

The tumor suppressor protein PTEN is a member of the protein tyrosine phosphatase family that can negatively regulate the serine/threonine kinase Akt to exert its tumor suppressor function.^26^ The protein phosphatase activity of PTEN can regulate cell migration, spreading and growth.^27^ PTEN is widely expressed in the mouse CNS and preferentially in neurons such as large pyramidal neurons.^28^ PTEN localizes to both the nucleus and cytoplasm of neuronal and glial cells.^28, 29, 30^ Significant progress has been made in investigating the broader role of PTEN in the brain. In addition to its normal functions such as control of neuronal migration^31, 32^ and neuronal size,^33, 34^ the PTEN protein is involved in pathological processes surrounding neuronal injury such as those associated with brain ischemia, neurological and mental disorders and drug addiction.^33, 34, 35, 36, 37, 38, 39^ Conditional inactivation of PTEN leads to behavioral abnormalities and neuropathological changes characterized by neuronal hypertrophy.^39^ Park *et al.*^40^ reported that modulation of the PTEN/mTOR pathway promotes axon regeneration in the adult CNS. To assess whether PTEN can be manipulated in iPS-derived MNs, PTEN was suppressed via siRNA in primary cultured and iPS-derived MNs. This resulted in decreased PTEN expression as assessed by immunofluorescence staining (Figures 3a and e) and western blotting (Figures 3b and f). Phosphorylation of Akt and Bad was increased following PTEN knockdown (Figures 3c, d, g and h). This is consistent with our previous report^15^ in primary cultured wild-type and SMA MNs.

### PTEN knockdown decreases AMPA receptor expression in primary cultured and iPS-derived MNs

**Figure 4:**
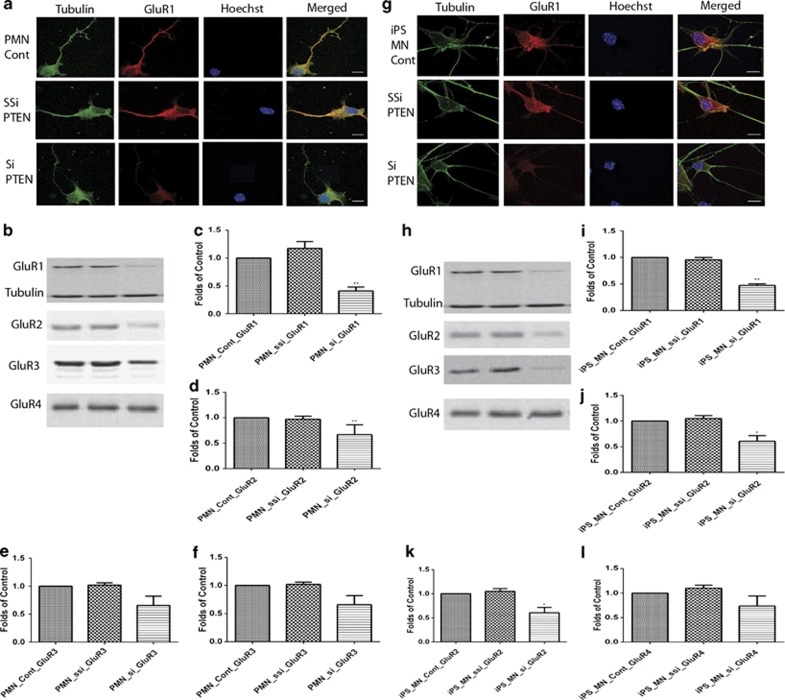
PTEN knockdown decreases AMPA receptor expression in primary cultured (**a**–**f**) and iPS-derived (**g**–**l**) motor neurons. Motor neurons were immunostained with anti-tubulin (green), Hoechst (nuclear staining in blue) and AMPA receptor (GluR1, red). Scale bar, 20 *μ*m. (**b**–**f** and **h**–**l**) Western blotting. PTEN knockdown decreases GluR1 (**c** and **i**) and GluR2 (**d** and **j**) but not GluR4 expression in both primary cultured and iPS-derived MNs. GluR3 decreases significantly in iPS-derived MNs (**k**) but not in primary cultured MNs (**e**). Results represent the mean+S.E.M. from four independent experiments. **P<0.01, tested by one-way ANOVA

The selective vulnerability of MNs to AMPA receptor-mediated excitotoxicity can be studied *in vitro* using purified MNs. These MNs are sensitive to AMPA- or kainate-induced excitotoxicity.^41^ AMPA- or kainate-induced MN death can be inhibited by NBQX, an AMPA receptor antagonist.^42^ In this study, we investigate the potential effects of PTEN on MN excitability in primary cultured and iPS-derived MNs. The first step was to assess whether PTEN knockdown has an effect on AMPA receptor expression. Here we showed that PTEN knockdown decreased GluR1 and GluR2 expression in primary cultured MNs (Figures 4a–c) and iPS-derived MNs (Figures 4g–j). GluR3 was decreased in iPS-derived MNs (Figure 4k), but not in primary cultured MNs (Figure 4e). There was no significant change in GluR4 expression in primary cultured MNs (Figure 4f) or iPS-derived MNs (Figure 4l) at 7 days post viral transfection.

### PTEN knockdown decreases AMPA-induced whole-cell currents in primary cultured and iPS-derived MNs

**Figure 5:**
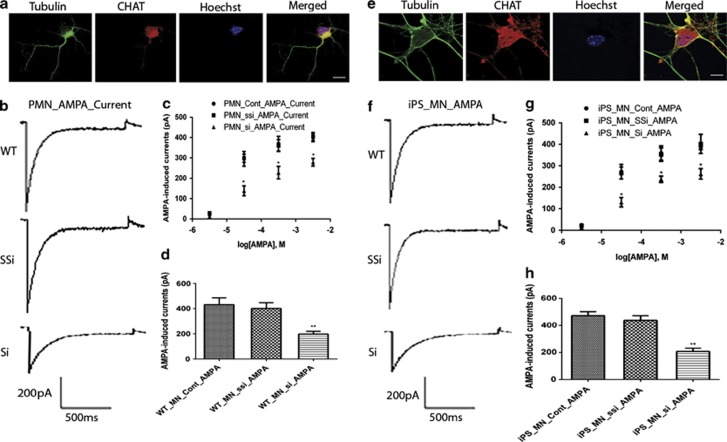
PTEN knockdown decreases AMPA-induced whole-cell currents in primary cultured (**a**–**d**) and iPS-derived (**e**–**h**) motor neurons. Primary cultured (**a**) and iPS-derived (**e**) motor neurons were immunostained with anti-tubulin (green), Hoechst (nuclear staining in blue) and ChAT (red). Scale bar, 20 *μ*m. (**b** and **f**) Representative current traces elicited by AMPA. (**c** and **g**) Dose response of AMPA-induced whole-cell currents were recorded in 20 mM extracellular Na+ at −60 mV, in response to AMPA concentrations ranging from 5 *μ*M to 5 mM. Each point represents the mean±S.E.M. from three cells (*P<0.05, **P<0.01, tested by one-way ANOVA). EC_50_ values estimated from fits to pooled data were 106 *μ*M for primary cultured motor neurons and 113 *μ*M for iPS-derived neurons. (**d** and **h**) Average peak AMPA-induced whole-cell currents were recorded in Na+-free extracellular solution containing 50 mM Ca^2+^ at −60 mV in primary cultured and iPS-derived motor neurons, evoked by AMPA 100 *μ*M (*n*=8). Columns represent the peak amplitudes of agonist-induced whole-cell currents. Results represent the mean+S.E.M. from four independent experiments, with 8 motor neurons of each (*P<0.05, **P<0.01, tested by one-way ANOVA)

Based on the decrease of expression of AMPA receptors following PTEN silencing, we next tested the effects of PTEN knockdown on the functional output of AMPA receptors in primary cultured and iPS-derived MNs. Whole-cell patch-clamp recording was performed to examine the effects of PTEN knockdown on the electrophysiological properties of primary cultured and iPS-derived MNs. Dose responses of AMPA-induced whole-cell currents were recorded in 20 mM extracellular Na+ at −60 mV, in response to AMPA concentrations ranging from 5 *μ*M to 5 mM. Each point represents mean±S.E.M. from three cells. Average peak amplitudes were 24±6 pA (5 *μ*M), 295±63 pA (50 *μ*M), 422±58 pA (500 *μ*M ) and 399±34 pA (5 mM) in control primary cultured MNs; 28±3 pA (5 *μ*M), 298±35 pA (50 *μ*M), 362±50 pA (500 *μ*M) and 404±34 pA (5 mM), respectively, in scramble siPTEN primary cultured MNs; 12±3 pA (5 *μ*M), 138±41 pA (50 *μ*M), 227±53 pA (500 *μ*M) and 278±32 pA (5 mM), respectively, in siPTEN primary cultured MNs. PTEN knockdown decreases AMPA-induced whole-cell currents in primary cultured MNs in a dose-dependent manner (*n*=3, *P<0.05, **P<0.01, tested by one-way ANOVA). Similarly, average peak amplitudes were 25±7 pA (5 *μ*M), 272±58 pA (50 *μ*M), 359±58 pA (500 *μ*M) and 389±22 pA (5 mM), respectively, in control iPS-derived MNs; 22±8 pA (5 *μ*M), 267±49 pA (50 *μ*M), 352±55 pA (500 *μ*M) and 402±76 pA (5 mM), respectively, in scramble siPTEN iPS-derived MNs; 13±3 pA (5 μM), 130±38 pA (50 *μ*M), 237±26 pA (500 *μ*M) and 262±42 pA (5 mM), respectively, in siPTEN iPS-derived MNs. PTEN knockdown decreases AMPA-induced whole-cell currents in iPS-derived MNs in a dose-dependent manner (*n*=3, *P<0.05, **P<0.01, tested by one-way ANOVA). The EC_50_ values estimated from fits to pooled data were 106 *μ*M for primary cultured MNs and 113 *μ*M for iPS-derived MNs (Figures 5d and h). Average peak AMPA-induced whole-cell currents were recorded in Na+-free extracellular solution containing 50 mM Ca^2+^ at −60 mV in primary cultured and iPS-derived MNs, evoked by AMPA 100 *μ*M (*n*=8). AMPA at 100 *μ*M was close to the EC_50_ from the dose-response recordings for primary cultured MNs (Figure 5c) and iPS-derived MNs (Figure 5g). Topical application with 100 *μ*M AMPA elicited a mean amplitude of whole-cell current in control (431±156 pA (*n*=8)), scramble siPTEN (401±126 pA (*n*=8)) and siPTEN (198±59 pA (*n*=8)), respectively, in primary cultured MNs and iPS-derived MN control (471±82 pA (*n*=8)), scramble siPTEN (437±96 pA (*n*=8)) and siPTEN (208±69 pA (*n*=8)), respectively. PTEN knockdown by siRNA significantly decreased AMPA-induced whole-cell currents in primary cultured and iPS-derived MNs (*P*<0.01).

### PTEN knockdown decreases AMPA-induced cell death in primary cultured and iPS-derived MNs

**Figure 6:**
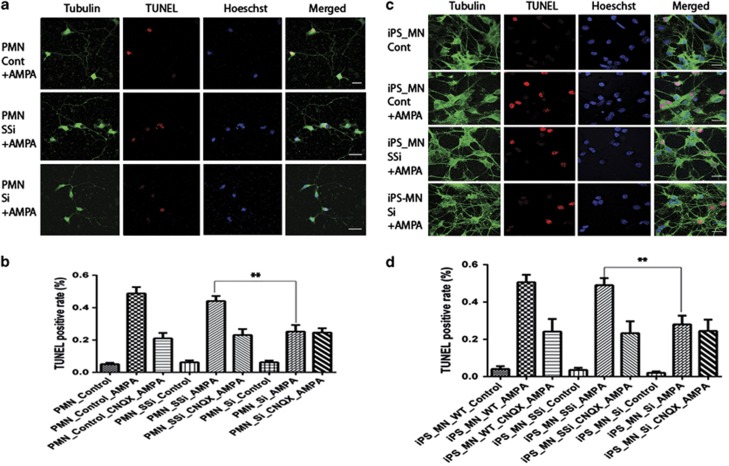
PTEN knockdown decreases AMPA-induced cell death in primary cultured and iPS-derived motor neurons. Primary cultured (**a**) and iPS-derived (**c**) motor neurons were immunostained with anti-tubulin (green), Hoechst (nuclear staining in blue) and TUNEL staining (red). Scale bar, 20 *μ*m. (**b** and **d**) Results represent the mean+S.E.M. from four independent experiments. **P<0.01, tested by one-way ANOVA

AMPA receptor trafficking plays a role in regulating excitatory synaptic strength and multiple growth factors, and hormones modulate this process.^43^ The effects of leptin on GluR1 trafficking involve an increase in PtdIns(3,4,5)P3 levels secondary to PTEN inhibition as the effects of leptin on surface GluR1 staining were associated with increased PtdIns(3,4,5)P3 levels and elevation in PtdIns(3,4,5)P3 levels occurred before altered GluR1 trafficking. Inhibitors of PI3-kinase attenuated the effects of leptin on surface GluR1 and PtdIns(3,4,5)P3 levels, consistent with PI3-kinase-dependent signaling underlying these effects.^44^ Alterations in PtdIns(3,4,5)P3 levels may promote structural rearrangement of the actin cytoskeleton that in turn influences the synaptic density of AMPA receptors.^45^ MNs are thought to be vulnerable to excessive AMPA receptor stimulation because of the presence of a high number of GluR2-lacking AMPA receptors. Low GluR2 levels yield AMPA receptors with a high Ca^++^ permeability.^46, 47, 48, 49^ A downregulation of GluR2 expression preceding neurodegeneration has never been observed. However, relatively low GluR2 expression is likely to contribute to the selective vulnerability of MNs to AMPA receptor stimulation. It has been recently reported that toxic factors released from glial cells expressing mt SOD1 contribute to the MN toxicity.^50, 51^ To determine whether downregulation of AMPA receptor expression can contribute to MN survival, we used the TUNEL (terminal deoxynucleotidyl transferase dUTP nick end labeling) assay to assess apoptotic cell death in primary cultured and iPS-derived MNs. Cells were treated with 50, 100, 200, 300, 400, 500 and 600 *μ*M (S)-AMPA to determine dose-response effects of AMPA exposure. In control primary cultured MNs, 5.1±1.6%, 8.1±2.3% (50 *μ*M), 11.5±2.1% (100 *μ*M), 21.0±3.7% (200 *μ*M), 48.4±9.7% (300 *μ*M), 59.1±9.7% (400 *μ*M), 66.6±12.5% (500 *μ*M) and 70.5±8.7% (500 *μ*M) TUNEL-positive cells were seen, respectively. Exposure to 300 *μ*M AMPA resulted in the death of ∼50% of the MNs and this was inhibited by the AMPA receptor inhibitor (cyano-7-nitroquinoxaline-2,3-dione (CNQX; Figure 6). PTEN knockdown significantly reduced cell death in primary cultured MNs (control 48.8%, scramble 44.1% and siPTEN 25.2%, respectively) and iPS-derived MNs (Control 50.5%, scramble 48.9% and siPTEN 27.9%, respectively) (Figure 6).

## Discussion

The aim of the present study was to determine whether PTEN knockdown has an effect on functional AMPA receptor expression and AMPA-mediated cell death in primary cultured and iPS-derived MNs. AMPA receptors are heteromeric complexes composed of various combinations of the four subunits: GLUR1 to GLUR4. The presence of the GLUR2 subunit in the assembled AMPAR determines its calcium permeability, and alternative flip or flop splicing of all subunits generates further diversity affecting the kinetic properties of AMPAR.^43, 44^ MNs are more susceptible to AMPA receptor-mediated death compared with other spinal neurons.^47, 48^ Deactivation and desensitization kinetics of AMPA receptors in MNs also resembled AMPA receptor kinetics in cerebellar Purkinje neurons.^47^ Activation of PI3-kinase is required for AMPA receptor insertion during long-term potentiation (LTP) of mEPSCs in cultured hippocampal neurons.^44^ Although the molecular control of AMPA receptor kinetics is complex and incompletely understood, subunit composition appears to be an important determinant of AMPA receptor desensitization.^52^

To assess functional AMPA receptors in primary cultured and iPS-derived MNs, we measured AMPA receptor-mediated current in both types of cells. PTEN knockdown decreased GluR1 and GluR2 expression and AMPA-induced whole-cell currents. Downregulation of GluR1 was more significant than of GluR2. Our findings appear to imply that the ratio of GluR1 and GluR2 changes following PTEN knockdown may cause variation in the AMPA receptor properies of calcium permeability and excitatory synaptic transmission. It has been reported that the antiglutamate drug, riluzole, is able to modulate AMPA receptors by reducing the kainate-induced currents in spinal MNs in a noncompetitive and a dose-dependent manner. AMPA is considered to play an important role in the selective death of MNs in ALS. A significantly different ratio between inhibitory and excitatory synapses was present in MNs from the SOD1^G93A^ mouse.^11^ The levels of the GluR2 AMPA subunit are decreased in spinal MNs before symptom onset in SOD1^G93A^ mice.^12^ Inefficient GluR2 Q/R site editing appears to be a disease-specific molecular alteration reported in sporadic ALS patients.^13^ At the level of the glutamatergic synapse, high concentrations of riluzole have been reported to inhibit glutamate release, attenuate excitatory amino acid receptor activation and decrease excitability of the postsynaptic cell membrane.^52^ The high concentrations required, however, are unlikely to be reached *in vivo* using clinically approved doses. AMPA receptors are glutamate-gated cation selective channels that mediate most fast excitatory synaptic transmission in the mammalian brain. AMPA receptor desensitization protects neurons against excitotoxic effects resulting from prolonged activation, as suggested by the pharmacological blockade of AMPAR desensitization that enhances excitotoxicity in neurons, including spinal MNs.^10, 46, 47, 48, 49, 52^ Here we report that PTEN inhibition decreases AMPA-induced cell death in primary cultured and iPS-derived MNs by downregulation of AMPA receptor expression and function assessed by AMPA-mediated whole-cell currents.

We have previously reported that oculomotor neurons resistant to the disease process in ALS show reduced AMPA-mediated inward calcium current compared with spinal MNs that are vulnerable to the disease process.^10^ These data implied that downregulation of AMPA receptor expression in spinal MNs might increase their resistance to exctitotoxic injury. We previously showed that PTEN depletion increased MN survival in models of SMA and ALS.^15, 16^ Here we show that PTEN inhibition decreases AMPA-mediated inward currents. Thus, PTEN depletion or inactivation may represent a therapeutic or neuroprotective strategy for AMPA receptor-mediated MN death.

It has been reported that reduction of kainate-induced currents by riluzole is consistent with its neuroprotective effects, because it hyperpolarizes the membrane and attenuates spike-firing rates, leading to reduced presynaptic glutamate release. However, riluzole has a limited effect in the clinical setting because the high concentrations required to achieve these effects are unlikely to be reached *in vivo* using clinically approved doses.^53^ PTEN silencing may achieve amelioration of AMPA receptor-mediated toxicity *in vivo*. Indeed, PTEN depletion has been shown to dramatically increase MN survival *in vivo*.^15^

We have observed that the results obtained from iPS-derived and primary cultured MNs are mostly but not always consistent; for example, GluR3 decreases significantly in iPS-derived MNs (Figure 4k) but not in primary cultured MNs (Figure 4e). We used the best PTEN shRNA sequence from multiple tested sequences for the lentiviral constructs for the transduction of primary MNs. This siRNA has been tested in neuronal cell lines^38, 54^ and MNs *in vitro*^16^ and *in vivo*.^15^ The *PTEN* siRNA virus we used for iPS-derived MNs was purchased from Santa Cruz Biotechnology Inc. (Dallas, TX, USA), which has been fully characterized by the company, and its efficiency of PTEN knockdown has been tested widely by various research groups.^55, 56^ As both the cells and the siRNA virus are different, it would be possible to have variation for GluR3 knockdown between primary cultured and iPS-derived MNs. This difference is unlikely to represent off-target effects. We have not seen significant PTEN knockdown using scrambled siRNA sequences and other siRNA sequences.^15, 16, 54, 55, 56^ We believe therefore that the PTEN knockdown is specific in both primary cultured and iPS-derived MNs.

In conclusion, we have shown for the first time that PTEN knockdown decreases the expression and activity of AMPA receptors in MNs. Modulation of AMPA receptors may underlie some of the neuroprotective effects of PTEN inhibition or reduced expression of the PTEN protein. In future work, we will use ALS patient fibroblasts to generate iPS-derived MNs and investigate the effect of PTEN knockdown on MN survival in patient iPS-derived MNs from specific subtypes of ALS patients. This may represent a useful strategy for drug screening to identify compounds that may modulate disorders involving neuronal hyperexcitability, including ALS.

## Materials and Methods

### Human fibroblast and Plat-A culture

Under human research subject and stem cell protocols as approved by the institutional review boards, we recruited a healthy 40-year-old Chinese female to donate a skin biopsy to be used in reprogramming studies and the production of pluripotent stem cell lines. Three other healthy controls were also used to generate iPSCs under ethical approval in the United Kingdom. The dermal fibroblasts were isolated by 0.25% trypsin (Gibco, Shanghai, China, 25200). HDFs and Plat-A cells were maintained in medium containing Dulbecco's modified Eagle's medium (DMEM; Gibco, 11965-092), 10% fetal bovine serum (FBS, Thermo, Shanghai, China), 1% nonessential amino acids (NEAA, Gibco, 11140-050) and 1% GlutaMAX (Gibco, 35050).

### Generation of hiPSCs from adult HDFs

Plat-A packaging cells were plated at 6 × 10^6^ cells per 100 mm dish and incubated overnight. After 1 day, the cells were transfected with retroviral vector pMXs (encoding Oct4, Sox2, Klf4 and c-Myc) using Lipofectamine 2000 transfection reagent (Gibco, GB11668-019). At 48 h after transfection, the medium was collected as the first virus-containing supernatant and replaced with new medium that was collected 24 h later as the second virus-containing supernatant. The virus-containing supernatants were filtered through a 0.45 *μ*m pore-size filter and supplemented with 10 *μ*g/ml polybrene (Sigma, St. Louis, MO, USA, H9268). Equal amounts of virus-containing supernatants containing each of the four retroviruses were mixed before transduction, transferred to the fibroblast dish and incubated overnight. Approximately 50 000 fibroblasts per well of a six-well plate were transduced twice over 48 h. Then, the transduced cells were passaged on plates containing irradiated MEFs. The medium was replaced with iPSCs medium, containing DMEM/F12 (Gibco, 11330), 20% knockout serum replacement (KSR, Gibco, 10828-028), 1% nonessential amino acids (NEAA, Gibco, 11140-050), 1% GlutaMAX (Gibco, 35050), 0.1 mM *β*-mercaptoethanol (Sigma) and 4 ng/ml bFGF (R&D, St. Louis, MO, USA). The first hiPSC colonies appeared ∼2 weeks later and they could be picked after 1–2 additional weeks of culture. Individual colonies were picked and either transferred into a single well of 12-well plates containing iPSC medium and irradiated MEFs. For passaging, hiPSCs were incubated with DMEM/F12 containing collagenase IV (1 mg/ml) at 37°C for 10–15 min. When colonies at the edge of the dish were dissociating from the bottom, the enzyme was removed and washed by iPSC medium without bFGF. Cells were collected by gently pipetting.

### RNA isolation and PCR analysis

Total RNA was isolated using the RNAsimple Total RNA Kit (Tiangen, Shanghai, China). Total RNA at 1 *μ*g was used for the reverse transcription reaction with the RevertAid First Strand cDNA synthesis kit (Fermentas, Beijing, China). The cDNA from MEFs was used as a negative control, whereas that from hESCs was used as a positive control. RT-PCR was performed using specific sequences:^57^ hOCT3/4-F 5′-GACAGG GGGAGGGGAGGAGCTAGG-3′, hOCT3/4-R 5′-CTTCCCTCCAACCAGTTGCCCCAAAC-3′ hSOX2-F 5′-GGGAAATGGGAGGGGTGCAAAAGAGG-3′, hSOX2-R 5′-TTGCGTGAGTGTGGATGGGATTGGTG-3′ hKLF4-F 5′-AGAAGCGACAGAATCAAA-3′, hKLF4-R 5′-GGACCTGGTATGTGGAGA-3′ hc-MYC-F 5′-GCGTCCTGGGAAGGGAGATCCGGAGC-3′, hc-MYC-R 5′-TTGAGGGGCATCGTCGCGGGAGGCTG-3′ hNANOG-F 5′-ACCAGGCACTACCGTAAACA-3′, hNANOG-R 5′-CCCTCATCGGGAAGACAG-3′ hLIN28-F 5′-GGATGTCTTTGTGCACCAGA-3′, hLIN28-R 5′-CTCCTTTTGATCTGCGCTTC-3′ REX1-F 5′-CAGATCCTAAACAGCTCGCAGAAT-3′, REX1-R 5′-GCGTACGCAAATTAAAGTCCAGA-3′ hGAPDH-F 5′-CAAGATCATCAGCAATGCCTCCTG-3′, hGAPDH-R 5′-GCCTGCTTCACCACCTTCTTGA-3′.

### Alkaline phosphatase staining and immunocytochemistry

Alkaline phosphatase staining was performed using the Alkaline Phosphatase Detection kit (Si Dan Sai, Shanghai, China, 1102). For immunocytochemistry, cells were fixed with phosphate-buffered saline (PBS) containing 4% paraformaldehyde (PFA) for 10 min at room temperature. After washing with PBS, the cells were treated with PBS containing 4% normal donkey serum (Jackson ImmunoResearch, West Grove, PA, USA, 017-000-121), 1% bovine serum albumin (BSA, Sigma) and 0.1% Triton X-100 for 45 min at room temperature. Primary antibodies included SSEA3 (1 : 100, Invitrogen, Paisley, UK), SSEA4 (1 : 100, Invitrogen), TRA-1-60 (1 : 200, Millipore, Billerica, MA, USA), TRA-1-81 (1 : 200, Millipore), OCT3/4 (1 : 100, Santa Cruz Biotechnology, Inc.), SOX2 (1 : 200, Millipore), TE-7 (1 : 200, Chemicon, Billerica, MA, USA), rabbit polyclonal antibodies against tubulin (1 : 500, Cell Signaling, Danvers, MA, USA), mouse monoclonal antibodies against tubulin (1 : 500, Millipore), rabbit monoclonal antibodies against PTEN (1 : 100, Cell Signaling), anti-glutamate receptor 1 (AMPA subtype) (1 : 100, Abcam, Cambridge, UK) and ChAT (1 : 100, Abcam). Secondary antibodies used were CF488A-conjugated donkey anti-goat IgG (1 : 800, Biotium, Hayward, CA, USA), CF488A-conjugated donkey anti-rat IgG (1 : 800, Biotium), CF488A-conjugated donkey anti-mouse IgG (1 : 800, Biotium), CF488A-conjugated donkey anti-rabbit IgG (1 : 800, Biotium), Cy3-conjugated donkey anti-rabbit and FITC-conjugated donkey anti mouse (1 : 300, Cell Signaling) and incubated at 4°C overnight. Cells were then washed three times with 1 × PBS. Mounted MNs were viewed using a Leica (Wetzlar, Germany) TCS SP5 confocal microscope.

### Teratoma formation

One million hWT-iPSCs cells were injected subcutaneously into the hind limb muscle of the NOD/SCID mouse (Jackson Laboratory, Bar Harbor, ME, USA). Paraffin sections of formalin-fixed teratoma specimens were prepared 6–8 weeks after injection, and analysis of H&E-stained tissue sections was performed for each specimen. All animal experiments were performed in accordance with the institutional guidelines.

### Bisulfite sequencing and karyotype analysis

Bisulfite treatment was performed using the EZ DNA Methylation-gold kit (ZYMO Research, Irvine, CA, USA), according to the manufacturer's recommendations. PCR primers are: out-forward, 5′-GTTAAGGTTAGTGGGTGGGATT-3′ out-reverse, 5′-ATCACCTCCACCACCTAAAAA-3′ in-forward, 5′-AGAGAGGGGTTGAGTAGTTTTTT-3′ in-reverse, 5′-ACCTCCACCACCTAAAAAAAAC-3′. Amplified products were cloned into pMD18-T Vector (Takara, Shiga, Japan). Ten randomly selected clones were sequenced with the M13 forward and M13 reverse primers for each gene. Chromosomal G-band analyses were performed at Tongji Hospital, (Shanghai, China).

### Viral vectors

A 19-nt sequence targeting mouse PTEN was subcloned in the pLVTHM genome vector (Addgene plasmid 12247, Cambridge, MA, USA) as previously described.^15^ Briefly, siPTEN sense oligonucleotide 5′-CGCGTCCCCGCCAAATTTAACTGCAGAGTTCAAGAGACTCTGCAGTTAAATTTGGCTTTTTGGAAAT and siPTEN antisense oligonucleotide 5′-CGATTTCCAAAAAGCCAAATTTAACTGCAGAGTCTCTTGAACTCTGCAGTTAAATTTGGCGGGGA were annealed and cloned into the *Ml*uI/*Cla*I-digested vector. Accordingly, scrambled siPTEN sense nucleotide 5′-CGCGTCCCCCGCAATATTCAATCGAGGATTCAAGAGATCCTCGATTGAATATTGCGTTTTTGGAAAT and ssiPTEN antisense nucleotide 5′-CGATTTCCAAAAACGCAATATTCAATCGAGGATCTCTTGAATCCTCGATTGAATATTGCGGGGGA were used to generate the control LV-ssiPTEN vector. The third generation of self-inactivating (SIN) lentiviral vector stocks involving four plasmids (pMD.2G, pCMVDR8.92, SIN-W-PGK and pRSV-Rev) were prepared by transient calcium phosphate transfection of the human embryonic kidney 293T cell line as previously described.^15, 57^ These vectors were pseudotyped with the vesicular stomatitis virus-G envelope protein. Viral titers were estimated using a FACS assay.^15^ For iPS-derived MNs, PTEN shRNA (h) Lentiviral vector containing three target-specific constructs that encode 19–25 nt (plus hairpin) shRNA designed to target human PTEN were purchased from Santa Cruz Biotechnology (sc-29459 and control shRNA Lentiviral Particles: sc-108080).

### MN culture

Cultures of embryonic spinal MNs were prepared as previously described.^15^ Briefly, the ventrolateral part of the E13 spinal cord was dissected and incubated at 37°C for 15 min in 0.1% trypsin in Hanks' balanced salt solution. After trituration, cells were plated on dishes precoated with anti-p75 nerve growth factor (NGF) receptor antibody (Abcam, ME20.4, ab8877) in Neurobasal (Gibco) for 30 min. The cells were washed three times with Neurobasal, and the attached cells were isolated from the plate with depolarizing solution (0.8% NaCl, 35 mM KCl) and collected in full media (Neurobasal supplemented with 2% horse serum, 2% B27 (Gibco), 0.1 mM *β*-mercaptoethanol (Sigma) and 1 × Glutamax (Gibco)). For staining, 2000 cells were plated on poly-DL-ornithine (Sigma) and mouse laminin (Invitrogen)-coated coverslips in four-well dishes (Greiner, Frickenhausen, Germany). For western blots, 100 000 cells were plated on each well of 12-well plate also coated with poly-ornithine and mouse laminin. MNs were transduced at 24 h after plating with multiplicity of infection (MOI) of 10. For all assays, brain-derived neurotropic factor was used at concentrations of 5 ng/ml and MNs were cultured at 37°C with 5% CO_2_. Medium was replaced after 24 h and then every 2 days. AMPA treatment for survival (TUNEL assay) and electrophysiology were carried out from day 7 onwards. Cells were treated with 50, 100, 200, 300, 400, 500 and 600* μ*M (S)-AMPA to determine dose-response effects of AMPA treatments. Of the neurons, 50% were TUNEL positive at 24 h following exposure to 300* μ*M AMPA for 1 h. For AMPA inhibition, 50 *μ*M CNQX (Sigma) was added to culture medium 1 h before MNs were exposed to 300 *μ*M (S)-AMPA (Sigma) for 1 h and then fresh medium was applied for 24 h before performing a TUNEL survival assay.^58^

### The iPS-derived MN differentiation

The iPS cells were maintained on gelatinized tissue-culture plastic in iPS media containing DMEM/F12 (Gibco, 11330), 20% knockout serum replacement (KSR, Gibco, 10828-028), 1% NEAA (Gibco, 11140-050), 1% GlutaMAX (Gibco, 35050), 0.1 mM *β*-mercaptoethanol (Sigma) and 4 ng/ml bFGF (R&D) at 37°C, 5% CO_2_. Medium was changed every 24 h. To generate MNs,^23, 24, 25^ iPS cells were passaged using dispase (1 mg/ml) and triturated into small cell clumps and placed into ultralow adherent culture dishes (Corning, Corning, NY, USA). For the first 3 days, cells were kept in suspension in iPS medium, to enhance single cell survival, and 20 ng/ml bFGF (Invitrogen) was added to enhance growth. At day 4, EBs were switched to neural induction medium (DMEM/F12 with L-glutamine; NEAA; penicillin/streptomycin; heparin, 2 *μ*g/ml; N2 supplement; Invitrogen). At day 6, all-*trans* retinoic acid (RA; 1 *μ*M; Sigma) was added. The same medium was changed with fresh RA daily. Hedgehog signaling was initiated on day 15 by application of SHH (100 ng/ml, R&D) and purmorphamine (PUR, 1 *μ*M). The same media was changed with freshly daily. At day 28, basal medium was changed to Neurobasal (Invitrogen), containing all previous factors, with the addition of 10 ng/ml of insulin-like growth factor 1 (IGF-1) and 1 *μ*M cAMP (Sigma). At day 35, EBs were dissociated with 0.05% trypsin (Invitrogen) and plated onto poly-lysine/laminin-coated dishes or coverslips. Plated neurons were cultured in the same medium as above with addition of ascorbic acid (200 ng, Sigma). Cells were transduced with human shRNA PTEN lentiviral vectors with MOI of 10 for 7 days before assessment. Cells were treated with 50, 100, 200, 300, 400, 500 and 600* μ*M (S)-AMPA to determine dose-response effects of AMPA treatment. Approximately 50% of the cells were TUNEL positive 24 h after exposure to 300* μ*M AMPA for 1 h. Exposure to AMPA with assays for survival (TUNEL assay) and electrophysiology were carried out from day 45 onwards. For AMPA inhibition, 50 *μ*M CNQX (Sigma) was added to culture medium 1 h before MNs were exposed to 300 *μ*M (S)-AMPA (Sigma) for 1 h and then fresh medium was applied for 24 h before performing a TUNEL survival assay.^58^

### Electrophysiology

Whole-cell electrophysiological experiments were recorded as previously described.^14^ Voltage clamp recordings were performed using an Axon Multi-Clamp 700B amplifier (Axon Instruments, Sunnyvale, CA, USA) using unpolished borosilicate pipettes placed at the cell soma. Pipettes had a resistance of 2–4 MΩ when filled with intracellular solution. Intracellular solution for AMPA- or kainate-induced currents consisted of 120 mM CsF, 3 mM MgCl_2_, 5 mM EGTA and 10 mM HEPES (pH adjusted to 7.25 with 12 mM CsOH). Cs+ in the pipette solution was included to block K^+^-dependent membrane conductance. Cells were accepted for study if a stable seal formed with a whole-cell resistance of at least 120 M Ω and a series resistance of <10 MΩ. Receptors were activated by focal perfusion of agonists from a micropipette with its tip located 30–50 *μ*m from the cell. Three cells were used for dose-response recordings for AMPA-induced whole-cell currents. Currents were recorded in 20 mM extracellular Na^+^ at −60 mV, in response to AMPA concentrations ranging from 5 *μ*M to 5 mM. The extracellular perfusion buffer consisted of 15.3 mM NaCl, 4.7 mM NaOH, 2 mM CaCl_2_, 10 mM HEPES, 10 mM glucose and 228 mM sucrose, pH 7.40. For the measurement of Ca^2+^ permeability, 100 *μ*M AMPA, close to the EC_50_ from the dose-response recordings, was used. All extracellular solutions were supplemented with MK- 801 (10 *μ*M), tetrodotoxin (1.0 *μ*M) and Cd^2+^ (100 *μ*M) to block NMDA receptors, voltage-gated Na^+^ channels and Ca^2+^ channels, respectively. Cells were held at a membrane potential of −60 mV. All recordings were performed at room temperature of 21–23°C. Current recordings were sampled onto an IBM-PC compatible computer using pClamp10 software (Axon). Data were filtered at 3 kHz and sampled at 20–40 kHz.

### TUNEL staining

Primary cultured or iPS-induced neurons were stained by the TUNEL technique (ApopTag@Red *In Situ* Apoptosis Detection Kit) according to the manufacturer's instructions (Millipore). MNs were fixed in 4% PFA in PBS at room temperature for 15 min. MNs were incubated in equilibration buffer for 1 h at room temperature. The buffer was then removed and samples were incubated in reaction buffer and TdT enzyme mix at 37°C for 90 min. The mix was then replaced with the stop buffer. Samples were kept at 37°C for 3 h. They were then washed in PBT and incubated with gentle shaking in blocking solution and anti-DIG-rhodamine mix overnight at 4°C. After that, rabbit anti-tubulin primary antibody (1 : 500; Cell Signaling) followed by CF488A-conjugated donkey anti-rabbit IgG (1 : 800, Biotium) was stained in green as described in the immunocytochemistry methods. Hoechst staining was used to stain nuclei in blue. Mounted MNs were viewed using a Leica TCS SP5 confocal microscope.

### Western blot analysis

Primary cultured or iPS-derived MNs were transduced with lentiviral vectors for 7 days and collected from the dishes. Protein extraction for western blotting was performed as described previously.^15, 16^ Primary antibodies, anti-mouse glyceraldehyde-3-phosphate dehydrogenase (GAPDH) antibody (1 : 5000; Calbiochem, Darmstadt, Germany), tubulin (1 : 5000; Millipore), anti-rabbit PTEN (1 : 1000; Cell Signaling), AKT (1 : 1000; Cell Signaling), Bad (1 : 1000; Cell Signaling), pAKT ser473 (1 : 1000; Cell Signaling), p-Bad ser155 (1 : 1000; Cell Signaling), Glutamate receptor 1 (AMPA subtype) (1 : 1000; Abcam), Glutamate receptor 2 (AMPA subtype) (1 : 1000; Cell Signaling), Glutamate receptor 3 (AMPA subtype) (1 : 1000; Cell Signaling), Glutamate receptor 4 (AMPA subtype) (1 : 1000; Cell Signaling), goat anti-rabbit or mouse HRP secondary antibodies (1 : 5000; Cell Signaling) were used.

### Statistical analysis

Power analysis was conducted using GPower 3.0 software (Oakland, CA, USA) and statistical analysis was performed using GraphPad Prism v5 (La Jolla, CA, USA). Statistical significance was determined by one-way analysis of variance (ANOVA) depending on the individual experiment as stated in the figure legends.

## Abbreviations

PTEN: phosphatase and tensin homolog deleted on chromosome 10
AMPA: *α*-amino-3-hydroxy-5-methyl-4-isoxazole propionic acid
iPSC: induced pluripotent stem cell
MN: motor neuron
ALS: amyotrophic lateral sclerosis
GluR: glutamate receptor
SOD1: superoxide dismutase 1
LCM: laser capture microdissection
C9orf72: chromosome 9 open reading frame 72
ADAR2: adenosine deaminase acting on RNA 2
SMA: spinal muscular atrophy
HDF: human dermal fibroblast
DMEM: Dulbecco's modified Eagle's medium
NEAA: nonessential amino acid
MEF: embryonic fibroblast
bFGF: fibroblast growth factor-basic
hESCs: human embryonic stem cell
ChAT: choline acetyltransferase
NGF: nerve growth factor
TUNEL: terminal deoxynucleotidyl transferase dUTP nick end labeling
CNQX: cyano-7-nitroquinoxaline-2,3-dione
PFA: paraformaldehyde
PBS: phosphate-buffered saline
RT-PCR: reverse transcription-PCR
GAPDH: glyceraldehyde-3-phosphate dehydrogenase
ANOVA: analysis of variance
